# Prevalence and Associated Factors of Work-Related Musculoskeletal Disorders Symptoms among Construction Workers: A Cross-Sectional Study in South China

**DOI:** 10.3390/ijerph20054653

**Published:** 2023-03-06

**Authors:** Yu-Chi Lee, Xinye Hong, Siu Shing Man

**Affiliations:** 1 College of Management and Design, Ming Chi University of Technology, New Taipei City 243303, Taiwan; 2 School of Design, South China University of Technology, Guangzhou 510006, China

**Keywords:** musculoskeletal disorders symptoms, Nordic musculoskeletal questionnaire, logistic regression, occupational health, construction

## Abstract

Statistics showed that work-related musculoskeletal disorders (WMSDs) are the leading cause of productivity loss in the construction industry. This study aimed to investigate the prevalence of WMSDs and associated factors among construction workers. A cross-sectional study was conducted among 380 construction workers in Guangdong Province, China. A demographic, work-related survey and the Nordic musculoskeletal questionnaire were used to collect the workers’ data. Descriptive statists and logistic regression were used for the data analysis. The results showed that the overall prevalence of WMSDs symptoms among the participants in any body region during the last 12 months was 57.9%. Neck (24.7%), shoulder (22.1%), upper back (13.4%), and lower back (12.6%) showed the highest prevalence of WMSDs. Age, exercise, work experience, work position, and level of fatigue after work were significantly associated with the prevalence of WMSDs symptoms in different body regions. The findings of this study showed that the prevalence of WMSDs symptoms among construction workers in south China is still high and is associated with different body areas compared to previous studies. The prevalence of WMSDs and risk-associated factors vary by country and region. This indicates that further local investigations are needed to propose specific solutions to improve the occupational health of construction workers.

## 1. Introduction

China has become the world’s second-largest economy through a vigorous economic development in the past three decades [1]. Meanwhile, due to the acceleration of urban construction, the construction industry has rapidly developed into a pillar industry of the national economy. In 2021, the total output value of China’s construction industry reached 29.307 trillion CNY, accounting for 25.63% of the gross domestic product [2], and the number of employed persons in construction enterprises was 53.67 million [3]. Along with the significant contribution of the construction industry to the economy and the vast population share, the occupational health of construction workers has become a primary research concern [4]. Studies have shown that work-related musculoskeletal disorders (WMSDs) are a common occupational health problem among construction workers [5] and pose a considerable threat to their quality of life and physical health [6].

Musculoskeletal disorders (MSDs) refer to pain and inflammation caused by injuries and disorders of soft tissues such as muscles, tendons, ligaments, joints, and cartilage. MSDs can affect almost all tissues, including nerves and tendons, involving the neck, shoulders, back, arms, and legs [7]. MSDs symptoms caused by movement during work activities are associated with WMSDs [8]. WMSDs are the most significant disability factor globally, with approximately 1.71 billion prevalent cases and 149 million years lived with disability in 2019 [9]. WMSDs not only affect the health of individuals and lead to disability but also have significant financial consequences for those affected in the form of sick leave and medical costs [10]. The US construction industry is estimated to incur over $400 million in workers’ compensation yearly due to WMSDs [11]. The work capacity of many construction workers is reduced by WMSDs, thereby leading to their early retirement [12]. 

As early as 2002, the International Labor Organization (ILO) included WMSDs in the list of international occupational diseases to protect workers’ rights. The symptoms of WMSDs in construction workers have been studied in many countries and regions. A previous study showed that the prevalence of self-reported WMSDs symptoms was high among construction workers in Boston [13]. Recent studies reported that the prevalence of WMSDs in construction workers in Pakistan [14], Southeastern Ethiopia [15], India [16], Iran [17], China [18] were 59.6%, 43.9%, 80%, 53.3%, and 23.4%, respectively. One study found that approximately 87% of construction workers in Korea reported WMSDs symptoms [19]. These findings highlight that the prevalence of WMSDs is high in the construction industry worldwide. 

Research showed that WMSDs are directly caused by physical working conditions, such as awkward postures, repetitive lifting, static force, carrying heavy objects, and vibrations [7]. Moreover, construction workers are chronically exposed to multiple physical risk factors (such as handling heavy manual materials, stretched body postures, and the constant use of machinery) [20,21], which may be the reason for the high prevalence of WMSDs in the construction industry. A growing body of evidence showed that psychosocial factors (such as the level of work fatigue, work stress, and social support) and individual factors (such as age, exercise, and education level) are predictors or risk factors for WMSDs [22]. Related research found that time pressures and job demands were associated with WMSDs among construction workers [23,24]. Thus, WMSDs are related to occupational and non-work factors (psychological factors and individual characteristics). Epidemiological studies have shown that the prevalence of WMSDs varies from industry to industry [25]. The diversity of work positions in the construction industry causes differences in WMSDs prevalence in the construction industry in different regions [5]. 

As the essence of WMSDs is complex, we need to conduct local research to establish protection systems. However, WMSDs have not been incorporated into China’s statutory occupational diseases list. As a result, these occupational health issues still receive little attention in China and are mainly concentrated in the manufacturing, medical and agricultural industries [8,26,27]. Several studies have included the construction industry when investigating WMSDs symptoms among Chinese workers. For instance, Jia et al. [18] indicated that the prevalence of WMSDs in the construction industry was 23.4% in China. Yi and Chan [28] reported that the prevalence of WMSDs among construction workers was 41% in Hong Kong, China. However, these studies were based on prevalence statistics and did not examine the factors associated with disease development in detail. Therefore, it is crucial to investigate WMSDs in the Chinese construction industry, which can provide more reliable and accurate data for the prevention and treatment of WMSDs. Thus, this study aimed to investigate the prevalence and associated risk factors for WMSDs in various body regions among construction workers and put forward recommendations for the prevention of WMSDs based on the results. The findings of this study suggest protective measures for construction workers to prevent occupational risks in the new environment, as well as improvement directions for the formulation of technical reforms in the construction industry. In addition, this study is expected to provide a theoretical foundation for establishing protective systems in countries lacking employee health and safety legislation.

## 2. Materials and Methods

### 2.1. Study Design and Participants 

This study was conducted as a cross-sectional survey from January to October 2022 among construction workers in Guangdong Province and explored the prevalence of WMSDs and their associated factors. The study participants were recruited from different large construction sites in the province. Construction workers with more than one year of work experience were included, while those with underlying diseases or accidents affecting the musculoskeletal system were excluded. All participants gave their informed consent before the investigation, and the institutional ethics committee approved the study protocol. A single population proportion formula [29] was used to estimate the sample size, assuming a 65% prevalence of WMSDs symptoms [16,30], a 5% margin of error, and a 95% confidence interval (CI). After adding a 10% for non-response rate, 385 construction workers were sampled using a random sampling method [31,32].

### 2.2. Questionnaire Design 

The study used a face-to-face questionnaire survey for data collection. A structured self-report questionnaire was designed by a panel of three ergonomic experts and two construction industry professionals. The self-report questionnaire consisted of three main parts: the first section collected demographic information, including gender, age, height, body weight, exercise habit, dominant hand, and education level. The second section concerned work-related characteristics, including work experience, work position, daily working hours, rest breaks, working days per week, job demands, and level of fatigue after work. The work-related questionnaire items were obtained based on previous studies [15]. The third section of the questionnaire was the Nordic Musculoskeletal Questionnaire (NMQ) [33], which was systematically translated into Chinese. The NMQ is a generalized questionnaire that collects information on WMSDs symptoms experienced in different body parts (neck, shoulders, upper back, elbows, hand/wrists, lower back, thigh/knees, ankles/feet) during the past 12 months. Deakin et al. [34] showed that the reliability of the questionnaire ranged from 0.77 to 0.98, and the validity ranged from 0.80 to 0.99. Moreover, some studies showed that the questionnaire maintained acceptable reliability and validity results when translated and adapted by different cultures and countries, including China [31]. 

### 2.3. Statistical Analysis

Statistical analysis was performed using SPSS 26.0 software (IBM Corporation, Armonk, NY, USA). A descriptive analysis was used to estimate the distribution of individual and work-related characteristics, and the Chi-square test was performed to evaluate correlations between the occurrence of WMSDs symptoms and the independent variables (individual and work-related factors). The prevalence of symptoms of WMSDs in construction workers is presented as a percentage. A binomial logistic regression analysis was performed to assess further the relationship between the occurrence of WMSDs symptoms in different body regions and the independent variables. In univariate logistic regression, the significance level of 5% (*p* < 0.05) was selected to insert the multivariate logistic regression model. Multivariate logistic regression analyses were performed using the stepwise selection method with forward-selected likelihood ratios, and the models were checked for fitness by the Hosmer–Lemeshow goodness-of-fit test. Finally, factors with *p* < 0.05 were considered statistically significant in the final model, and the strength of association with 95% CI was used for the adjusted odds ratio (AOR).

## 3. Results

### 3.1. Individual and Work-Related Characteristics

A total of 385 construction workers were surveyed, with a valid questionnaire response rate of 98.7% (*n* = 380). The age of the respondents ranged from 20 to 57 years (mean = 38.67 years, standard deviation [SD] = 9.05 years). The participants included 337 male and 43 female. The average height and weight (SD) of the respondents were 167.48 (6.20) cm and 63.59 (9.62) kg, respectively. The majority (75.53%, *n* = 287) of the respondents had a body mass index (BMI) in the normal range. Less than half of the respondents (43.1%, *n* = 164) usually exercised. The majority (82.10%, *n* = 312) of the respondents’ dominant hand was the right hand. Moreover, 60.79% (*n* = 231) of the participants attended a secondary school and above.

The respondents’ average work experience in construction-related jobs was 3.46 (3.92) years. For the work position, 44 (11.6%) were ironworkers, 57 (15%) were concreters, 68 (17.9%) were bricklayers, 76 (20%) were interior decorators, and 135 (35.5%) were general workers. Ironworkers use tools and machinery to descale, straighten, connect, cut, shape, and install rebar skeletons. The tasks performed by bricklayers are manual material handling and concrete work. Bricklayers can construct walls, floors, pavers, and other structures from brick, stone, and other masonry units with mortar or other binding materials. The work of interior decorators (including plasterers, carpenters, and plumbers) involves painting a site’s walls, installing electrical and water circuits and various pipes, and maintaining the walls and roofs for renovation [35]. General workers (surveyors, elevator drivers, laborers) are involved in sieving sand and gravel, mixing cement, carrying mortar, and some mechanical control [16,36]. The average working days per week and daily work hours were 6.45 (0.70) days and 8.5 (0.89) hours, respectively, with a break of approximately one hour during work. Some of the respondents (172, 45.26%) perceived their work content to be biased toward physical demands, while others (195, 51.3%) perceived it as characterized by both physical and mental demands. The majority of the respondents (70%, *n* = 266) reported a low fatigue level after work. Table 1 details the participants characteristics. 

In addition, the Chi-square test indicated that BMI (χ^2^ = 5.675, *p* = 0.017), exercise (χ^2^ = 17.51, *p* < 0.001), dominant hand (χ^2^ = 27.563, *p* < 0.001), work experience (χ^2^ = 10.934, *p* = 0.004), work position (χ^2^ = 37.058, *p* < 0.001), rest (χ^2^ = 9.923, *p* = 0.002), and level of fatigue after work (χ^2^ = 34.428, *p* < 0.001) were correlated with the occurrence of WMSDs among the workers.

### 3.2. Prevalence of WMSDs Symptoms

Overall, 57.9% (*n* = 220) of the workers reported varying degrees of WMSDs in at least one area of their body in the past 12 months. The highest prevalence of WMSDs symptoms among construction workers occurred in the neck (24.7%), followed by the shoulder (22.1%), upper back (13.4%), and lower back (12.6%). Regarding the different work positions, the highest and lowest prevalence of WMSDs occurred in the interior decorators (78.9%) and general workers (39.3%), respectively. Furthermore, the body region with the highest prevalence of WMSD in interior decorators and general workers was the neck. The shoulders were found to be the highest diseased area among concreters and bricklayers. Both neck and shoulders were the body areas with the highest prevalence of WMSD in ironworkers. Table 2 details the prevalence of WMSDs symptoms among the respondents.

### 3.3. Relationship between Associated Factors and WMSDs Symptoms in Different Body Regions

The results of the univariate logistic regression analysis are presented in Table 3. Age, exercise, smoking, work experience, work position, rest break, job demand, and level of fatigue after work were significantly associated with at least one body region with higher prevalence of WMSD (neck, shoulder, upper back, and lower back). Furthermore, to analyze the relationships between possible causing factors and the development of WMSDs symptoms in different body parts, the statistically significant variables mentioned in Table 3 were subsequently applied as potential factors in multivariate logistic regression. 

Table 4 showed that age, exercise, work experience, work position, and level of fatigue after work remained significant risk factors for neck, shoulder, upper back, and lower back pains. Participants older than 40 years had 9.89 times higher odds of developing upper back pain when compared to participants aged ≤25 years (AOR 9.89; 95% CI 1.25–78.20; *p* < 0.05). Moreover, participants who did exercise had 53%, 58%, and 59% less likely odds of developing shoulder (AOR 0.47; 95% CI 0.27–0.83; *p* < 0.01), upper back (AOR 0.42; 95% CI 0.21–0.85; *p* < 0.05), and lower back (AOR 0.41; 95% CI 0.21–8.51; *p* < 0.05) pain than those who never did exercise. Construction workers who had worked for 6–15 years were 3.20 times more likely to be at risk for shoulder pain than those who had worked for ≤5 years (AOR 3.20; 95% CI 1.71–6.00; *p* < 0.001). Regarding the work position, interior decorators were 4.55 times at higher odds of developing WMSDs in the neck than general workers (AOR 4.55; 95% CI 2.38–8.67; *p* < 0.001). Ironworkers (AOR 3.81; 95% CI 1.57–9.22; *p* < 0.01), concreters (AOR 3.01; 95% CI 1.34–6.73; *p* < 0.01), and interior decorators (AOR 2.92; 95% CI 1.35–6.32; *p* < 0.001) had higher odds of shoulder WMSDs symptoms than general workers. Ironworkers (AOR 3.29; 95% CI 1.27–8.51; *p* < 0.05) and interior decorators (AOR 3.68; 95% CI 1.48–9.12; *p* < 0.001) had higher odds of lower back WMSDs symptoms than general workers. The level of fatigue after work was also a potential factor for WMSDs symptoms in the shoulders and upper back. Workers who felt low had 3.68 (AOR 3.68; 95% CI 1.20–11.35; *p* < 0.05) and 3.68 (AOR 3.68; 95% CI 1.05–12.97; *p* < 0.05) times higher odds of WMSDS symptoms. In addition, the explanatory power of the models for the neck, shoulders, and lower back was generally high, with pseudo R^2^ [Nagelkerke] values of 0.116, 0.181, and 0.123, respectively. The model’s explanatory power for the lower back was low, with a pseudo R^2^ [Nagelkerke] value of 0.082. Moreover, the Hosmer–Lemeshow test results showed that the predicting models’ *p* values were 1.00, 0.315, 0.628, and 0.982 for the neck, shoulders, upper back, and lower back, which indicated that the models were adequately calibrated.

## 4. Discussion

In this study, we investigated the presence of WMSDs symptoms among construction workers in south China and established potential risk factors associated with WMSDs. The overall prevalence of self-reported WMSDs symptoms among construction workers was 57.9% in this study, which was higher than in studies conducted in Hongkong (41%) and mainland China (23.4%) [18,28]. However, the result differed from previous research in Korea (87%), Southeastern Ethiopia (43.9%), and India (80%) [15,16,19]. The different participant samples and data collection techniques in each study can explain the differences in symptom prevalence across these studies. For example, this study and Lette [15] used face-to-face interviews, and part of the research by Jia et al. used online questionnaires [18]. Moreover, the samples’ jobs in different studies were different. For example, the sample in this study was mainly composed of construction workers in five work positions. Chakraborty et al. [16] investigated six occupational groups, and Lette [15] investigated seven; Yi and Chan reported WMSDs of construction workers under the 14 different work positions [28]. The findings of this study are consistent with previously proposed prevalence rates of 25–92% for construction workers [22], while the exact prevalence rates may vary by country, state, and region. 

This study found that the most common body region of WMSDs symptoms was the neck (24.7%), followed by shoulders (22.1%), upper back (13.4%), and lower back (12.6%), among construction workers. The results are consistent with previous research showing that WMSDs in the construction industry mainly occur in the neck, shoulders, and lower back [18]. However, previous studies indicated that the highest prevalence of WMSDs in construction workers was in the lower back [14]. This is inconsistent with our results, and the possible reason may be the task content and working environment. Lette et al. [37] and Mustapha et al. [37] reported that construction workers involved in manual handling and carrying activities were more likely to suffer from lower back pain [37]. In this study, mechanical equipment, such as lifters, was used for carrying tasks due to the technology development and health concerns previously raised.

The results of this study showed that the prevalence of WMSDs varied significantly among the body parts of workers in different positions in the construction industry. Nazri et al. [38] showed that bricklayers had the highest prevalence of WMSDs in the shoulder, and Deros et al. [30] found that decorators, among Malaysian construction workers, had the highest prevalence of WMSDs in the neck. Lop et al. [38] concluded that concreters were more likely to have WMSDs in the shoulders. These results are consistent with the present study. Moreover, for reinforcement and general workers, the areas with a higher prevalence of WMSDs among the subjects in this study were mainly the upper limbs. In contrast, Chakraborty et al. [16] indicated that the body areas with pain among reinforcement workers in India were the lower back, followed the upper limbs. This difference may be due to differences in the job content and work process of construction workers in different regions. 

The findings of this study revealed that age, exercise, work experience, work position, and level of fatigue after work significantly affected the occurrence of WMSDs symptoms in at least one body region (neck, shoulders, upper back, and lower back). Workers aged >40 years were more likely to develop back WMSDs than those aged <25 years. Other studies support this result, as the body’s biological structures degenerate with age, especially those related to bone and muscle [39]. As a result, the functional capacity of the connective tissue and muscle strength decrease with age, making it more likely for upper back WMSDs to occur [40]. In addition, work experience was an essential factor affecting the development of WMSD symptoms in the shoulders. Due to the cumulative effect of WMSDs, the prevalence of shoulder WMSDs was higher in workers with 5–15 years of work experience than in workers with less than 5 years of work experience. Similar results were reported in previous studies where workers with more work experience were associated with increased upper back WMSDs due to cumulative exposure to WMSDs risk factors over time [41]. Moreover, workers who do not engage in physical activity are more likely to develop shoulder, upper back, and lower back WMSDs than those who do. Possible reasons for this are that physical activity increases muscle strength and flexibility, improves the musculoskeletal system, and helps to alleviate the symptoms of WMSDs [42,43]. Furthermore, there was a positive correlation between the level of fatigue after work and the symptoms of WMSDs in the shoulder and upper back. The higher the perceived level of fatigue, the greater the likelihood of the occurrence of symptoms of WMSDs, a that is consistent with previous studies [44,45]. Excessive physical exertion can affect the musculoskeletal system by causing fatigue and muscle stiffness and tightness. 

Notably, the work position was also found to be significantly associated with the occurrence of WMSDs in the neck, shoulder, and lower back. In particular, interior decorators were more likely than general workers to develop WMSDs in the neck. Ironworkers, concreters, and interior decorators were more likely to develop shoulder WMSDs than general workers. Interior decorators were more likely to be affected than general workers in the lower back. The prevalence of WMSDs showed significant differences depending on the type of work [36,46,47]. This difference can result from job requirements and work posture. Interior decoration involves static postures above shoulder height, which poses a significant risk of neck and shoulder MSDs [48]. In addition, the work of ironworkers, concreters, and interior decorators involves more hand-intensive tasks that are more likely to increase the risk of developing WMSDs in the upper extremities [49]. Moreover, concrete workers and interior decorators have a higher body load than general workers, and interior decorators perform more work involving floors than general workers, which leads to a higher incidence of back problems among concrete workers and interior decorators [50]. 

With the rapid growth of China’s economy, the construction industry is changing in terms of technology and equipment. In this study, the results showed that the highest prevalence of WMSDs among construction workers was mainly in the neck and shoulders, not the lower back. This result may be related to the fact that new equipment such as passive exoskeletons [51] used in the construction industry has reduced manual lifting work and the load on workers’ lower back. However, the overall prevalence of WMSDs among construction workers remains high. Further evaluation and improvement of the new equipment are needed. The occurrence of symptoms of WMSDs is associated with personal and characteristic job factors. Therefore, targeted ergonomic interventions are needed to appropriately reduce the risk of WMSDs in construction industry workers. For example, construction workers should properly rest and actively relax their muscles, especially interior decorators and cement workers with high prevalence of lower back and shoulder diseases. At the same time, some auxiliary equipment can be used to reduce workers’ body overload and limb overstretching. For example, interior decorators are recommended to set up automatic lifts to assist in completing their work under different heights. In addition, given a large number of construction workers in China and the high incidence of MSDs among construction workers in China, it is recommended that a national surveillance system should be established to record the MSDs of construction workers. This can protect workers’ health and related rights by recording medical records, injury records, or employer injury reports.

Several limitations of this study should be highlighted. Firstly, the data obtained for the current study were retrospective and self-reported and could be subject to recall bias, perhaps affecting the findings. Secondly, as the present study was cross-sectional, it was a one-time measurement of exposure and outcomes, and the causal relationships could not be determined [52]. Thirdly, this study was conducted at several construction sites in China, so the generalizability of the findings may be limited. Fourthly, this study did not investigate further work characteristics (such as working posture, physical loading), which are critical for estimating workers’ exposure to ergonomic hazards. Fifthly, due to selection bias or confounding, there may be a ‘healthy worker effect’ confounding the relationships between certain risk factors and outcomes in this study. Thus, it is recommended that future studies should use specialized techniques (such as marginal structural models, G-null tests, Monte Carlo G-computation algorithms, or G-estimation methods) to overcome this issue [53]. Therefore, we suggest that future studies include adequate sample sizes from different regions and be conducted using a more prospective approach to refine the findings.

## 5. Conclusions

This study explored the prevalence of symptoms and associated risk factors for WMSDs in construction workers in southern China. The NMQ was used to determine the prevalence of WMSD symptoms, and logistic regression was used to analyze the relationship between pain in various parts of the body and risk factors. The prevalence of overall discomfort among construction workers in the last 12 months was 57.9%, and the neck (24.7%) was the most common site of pain, followed by the shoulders (22.1%), upper back (13.4%), and lower back (12.6%). The variation in the WMSD prevalence results among construction workers across studies may be due to differences in work characteristics and environments. Additionally, this study found that age, exercise, work experience, work position, and level of fatigue after work had a statistically significant effect on discomfort in at least one area, among neck, shoulders, upper back, and lower back. We recommend that construction workers stretch reasonably and take proper rest during work to relax their muscles and actively restore their physical conditions. At the same time, some auxiliary equipment can be added to reduce the overload of the workers’ body and the excessive extension of their limbs. The results of this cross-sectional analysis can be used as a reference, and more attention should be paid to the specific tasks performed by the construction workers. 

## Figures and Tables

**Table 1:** Individual and work-related characteristics and their associations with musculoskeletal disorders symptoms among construction workers in Guangdong Province, 2022 (*n* = 380).

Variables	*n*	Presence of WMSDs Symptoms	χ^2^	*p*-Value
Gender				
Men	337	57.0%	1.037	0.308
Women	43	65.1%		
Age				
≤25 years	38	71.1%	3.067	0.216
26–40 years	174	55.7%		
>40 years	168	57.1%		
Body mass index			5.675	0.017 *
Normal (18.5–24.9 kg/cm^2^)	287	61.3%		
Abnormal	93	47.3%		
Exercise				
Never	216	67.1%	17.51	<0.001 ***
Occasionally and more	164	45.7%		
Dominant hand				
Right	312	64.1%	27.563	<0.001 ***
Left	68	29.4%		
Education level				
Primary school	96	63.5%	1.723	0.422
Middle school	231	56.3%		
High school	53	54.7%		
Work experience				
≤5 years	139	64%	10.934	0.004 **
6–15 years	180	59.4%		
>15 years	61	39.3%		
Work position				
Ironworker	44	70.5%	37.058	<0.001 ***
Concreter	57	64.9%		
Bricklayer	68	57.4%		
Interior decorator	76	78.9%		
General worker	135	39.3%		
Daily working hours				
≤8 h	217	55.3%	1.398	0.237
>8 h	163	61.3%		
Working day pre week				
≤5 days	17	70.6%	1.176	0.278
>5 days	363	57.3%		
Rest break				
Never	98	53.2%	9.923	0.002 **
Occasionally and more	282	65.1%		
Job demands				
Physical	172	60.5%	2.556	0.279
Mental	13	38.5%		
Both	195	56.9%		
Level of fatigue after work				
Never	46	26.1%	34.428	<0.001 ***
Low	266	58.6%		
Moderate	60	81.7%		
High	8	37.5%		

* WMSDs, work-related musculoskeletal disorders; * *p* < 0.05; ** *p* < 0.01; *** *p* < 0.001.

**Table 2:** Prevalence of work-related musculoskeletal disorders symptoms in different body regions among construction workers in various work positions in Guangdong Province, 2022.

Body Regions	Any Position [*n* (%)]	Ironworker [*n* (%)]	Concreter [*n* (%)]	Bricklayer [*n* (%)]	Interior Decorator [*n* (%)]	General Worker [*n* (%)]
Neck	94 (24.7%)	13 (29.5%)	12 (21.1%)	10 (14.7%)	37 (48.7%)	22 (16.3%)
Shoulders	84 (22.1%)	13 (29.5%)	18 (31.6%)	16 (23.5%)	21 (27.6%)	16 (11.9%)
Upper back	51 (13.4%)	4 (9.1%)	13 (22.8%)	8 (11.8%)	9 (11.8%)	17 (12.6%)
Elbows	34 (8.9%)	6 (13.6%)	10 (17.5%)	4 (5.9%)	8 (10.5%)	6 (4.4%)
Hands/wrists	39 (10.3%)	6 (13.6%)	13 (22.8%)	9 (13.2%)	6 (7.9%)	5 (3.7%)
Lower back	48 (12.6%)	4 (9.1%)	11 (19.3%)	10 (14.7%)	14 (18.4%)	9 (6.7%)
Thighs/knees	36 (9.5%)	4 (9.1%)	10 (17.5%)	4 (5.9%)	13 (17.1%)	5 (3.7%)
Ankles/feet	20 (5.3%)	2 (4.5%)	8 (14.0%)	3 (4.4%)	3 (3.9%)	4 (3.0%)
Any region	220 (57.9%)	31 (70.5%)	37 (64.9%)	39 (57.4%)	60 (78.9%)	59 (39.3%)
Sample size	380	44	57	68	76	135

**Table 3:** Factors associated with WMSDs in different body parts in construction workers in Guangdong Province, 2022: univariate logistic regression.

Variables	Neck	Shoulders	Upper Back	Lower Back
	OR [95% CI]	OR [95% CI]	OR [95% CI]	OR [95% CI]
Age				
≤25 years	Ref	Ref	Ref	Ref
26–40 years	0.88 [0.41–1.92]	1.60 [0.58–4.41]	4.27 [0.55–33.00]	2.08 [0.46–9.36]
>40 years	0.69 [0.32–1.52]	2.41 [0.89–6.57]	8.71 [1.15–65.84] *	3.60 [0.82–15.82]
Exercise				
Never	Ref	Ref	Ref	Ref
Occasionally and more	1.03 [0.64–1.64]	0.48 [0.28–0.80] **	0.40 [0.21–0.79] **	0.45 [0.23–0.87] *
Work experience				
≤5 years	Ref	Ref	Ref	Ref
6–15 years	0.82 [0.50–1.35]	3.24 [1.78–5.89] ***	1.03 [0.54–1.99]	1.86 [0.93–3.73]
>15 years	0.31 [0.13–0.74] **	1.58 [0.69–3.61]	1.16 [0.49–2.76]	1.06 [0.38–2.93]
Work position				
General worker	Ref	Ref	Ref	Ref
Ironworker	2.15 [0.98–4.76]	3.12 [1.36–7.17] **	0.69 [0.22–2.19]	1.40 [0.41–4.79]
Concreter	1.37 [0.63–3.00]	3.43 [1.60–7.37] **	2.05 [0.92–4.57]	3.35 [1.30–8.60] *
Bricklayer	0.89 [0.39–2.00]	2.29 [1.06–4.92] *	0.93 [0.38–2.27]	2.41 [0.93–6.26]
Interior decorator	4.87 [2.57–9.25] ***	2.84 [1.38–5.86] **	0.93 [0.39–2.21]	3.16 [1.30–7.71] *
Rest				
Never	Ref	Ref	Ref	Ref
Occasionally and more	0.54 [0.33–0.90] *	1.79 [0.97–3.31]	1.15 [0.58–2.30]	2.21 [0.96–5.11]
Job demands				
Physical	Ref	Ref	Ref	Ref
Mental	0.24 [0.03–1.92]	0.47 [0.10–2.20]	0.38 [0.05–3.02]	1.94 [0.50–7.60]
Both	0.98 [0.61–1.56]	0.55 [0.33–0.90] *	0.49 [0.27–0.91] *	0.82 [0.44–1.54]
Perceived work fatigue				
Never	Ref	Ref	Ref	Ref
Low	2.34 [0.95–5.75]	2.99 [1.03–8.69] *	2.03 [0.60–6.91]	2.24 [0.66–7.61]
Moderate	2.64 [0.95–7.35]	4.87 [1.52–15.54] **	3.58 [0.95–13.55]	2.21 [0.55–8.83]
High	2.22 [0.36–13.66]	3.50 [0.52–23.42]	8.6 [1.35–54.64] *	2.05 [0.19–22.57]

* OR, odd ratio; CI, confidence interval; WMSDs, work-related musculoskeletal disorders; Ref, reference group; * *p* < 0.05; ** *p* < 0.01; *** *p* < 0.001.

**Table 4:** Final model for the factors associated with WMSDs in different body parts in construction workers in Guangdong Province, 2022: multivariate logistic regression.

Body Region	Variables	AOR [95% CI]	R^2^
Neck	Work position		0.116
	General worker	Ref	
	Ironworker	2.22 [1.00–4.50]	
	Concreter	1.49 [0.67–3.28]	
	Bricklayer	0.92 [0.41–2.10]	
	Interior decorator	4.55 [2.38–8.67] ***	
Shoulders	Exercise		0.181
	Never	Ref	
	Occasionally and more	0.47 [0.27–0.83] **	
	Work experience		
	≤5 years	1.00	
	6–15 years	3.20 [1.71–6.00] ***	
	>15 years	1.76 [0.73–4.22]	
	Work position		
	General worker	Ref	
	Ironworker	3.81 [1.57–9.22] **	
	Concreter	3.01 [1.34–6.73] **	
	Bricklayer	2.07 [0.93–4.62]	
	Interior decorator	2.92 [1.35–6.32] ***	
	Perceived work fatigue		
	Never	Ref	
	Low	3.68 [1.20–11.35] *	
	Moderate	5.11 [1.53–17.56] **	
	High	4.54 [0.59–35.06]	
Upper back	Age		0.123
	≤25 years	Ref	
	26–40 years	4.91 [0.61–39.23]	
	>40 years	9.89 [1.25–78.20] *	
	Exercise		
	Never	Ref	
	Occasionally and more	0.42 [0.21–0.85] *	
	Perceived work fatigue		
	Never	Ref	
	Low	3.68 [1.05–12.97] *	
	Moderate	4.35 [1.13–16.69] *	
	High	17.73 [2.38–131.98] **	
Lower back	Exercise		0.082
	Never	Ref	
	Occasionally and more	0.41 [0.21–0.82] *	
	Work position		
	General worker	Ref	
	Ironworker	1.45 [0.42–5.01]	
	Concreter	3.29 [1.27–8.51] *	
	Bricklayer	2.33 [0.89–6.07]	
	Interior decorator	3.68 [1.48–9.12] ***	

* AOR, adjust odd ratio; CI: confidence interval; WMSDs, work-related musculoskeletal disorders; Ref, reference group; R^2^, Nagelkerke determination coefficient; * *p* < 0.05; ** *p* < 0.01; *** *p* < 0.001.

## Data Availability

The data presented in this study are available on request from the corresponding author. The data are not publicly available due to privacy reasons.
